# Attitudes of medical professionals towards high-risk suicidal patients

**DOI:** 10.3389/fpsyt.2025.1654240

**Published:** 2025-11-11

**Authors:** Tomonori Kashiwagi, Kenji Narita, Yusuke Tsuyama, Naohiro Yonemoto, Ryutaro Ishibashi, Chiaki Kawanishi

**Affiliations:** 1 Department of Neuropsychiatry, Sapporo Medical University Graduate School of Medicine, Sapporo, Hokkaido, Japan; 2 Department of Biostatistics, University of Toyama, Toyama, Japan; 3 Department of Public Health, Juntendo University Graduate School of Medicine, Bunkyo-ku, Tokyo, Japan

**Keywords:** attitude toward suicide, nurse, social worker, fire and emergency services personnel, suicide prevention

## Abstract

**Aim:**

Suicide is a serious global public health challenge recognised as a high priority by the World Health Organization. Knowledge and attitudes of healthcare professionals and first responders play a critical role in suicide prevention. This study aimed to clarify attitudes and influencing factors among paramedics, nurses, and social workers in Japan.

**Methods:**

We conducted a cross-sectional survey between 2015 and 2022 using a self-administered paper questionnaire. The Japanese version of the Attitudes Toward Suicide scale assessed six factors using a five-point Likert scale. A linear regression model was used to analyse variations associated with age, gender, profession, and experience.

**Results:**

Among 685 respondents, 443 provided valid responses (paramedics: 34.8%, nurses: 38.8%, social workers: 26.4%). Paramedics were more likely than social workers to regard suicide as an unjustified act (β = 0.474, 95% CI: 0.167–0.780, P = 0.003) and as a threat (β = 0.450, 95% CI: 0.142–0.757, P = 0.004), and less likely to think suicide can happen to anyone (β = −0.640, 95% CI: −0.855–−0.425, P < 0.001). Nurses regarded suicide as a threat (β = 0.394, 95% CI: 0.134–0.653, P = 0.003) and as an impulsive act (β = 0.220, 95% CI: 0.024–0.416, P = 0.028).

**Conclusion:**

Although we found differences in attitudes towards suicide between health professionals, there were also many commonalities. By holding workshops with a wide range of professionals, and deepening mutual understanding, it may be possible to implement seamless suicide prevention measures.

## 1 Introduction

Suicide has long been considered one of the most serious public health challenges globally. Suicide rates vary significantly among countries worldwide, with each nation and region’s cultural context influencing suicide patterns. For example, Japan has long been a country with a high suicide rate, and it possesses a historical cultural environment that tolerates suicide (1, 2). It has been noted that such community attitudes influence suicide rates, and that changes in community attitudes can affect changes in suicide rates (3, 4).

The World Health Organization recognizes suicide prevention as a global priority and has raised awareness about the universal nature of suicide issues and the fundamentals of addressing them (5). Furthermore, the World Health Organization has developed and published a suicide prevention guide for various interpersonal support professionals and those involved in the frontline response to suicide-related behaviors, including first responders such as fire and emergency services personnel, primary health care workers, general physicians, and others (6–11). While practitioners involved in suicide prevention efforts are also likely influenced by community attitudes, disseminating knowledge about suicide issues, suicide prevention concepts, and skills to these practitioners is crucial for preventing suicide.

Previous studies have shown that the knowledge and attitudes of interpersonal support workers are important in suicide prevention, and that workers’ attitudes are related to their intervention skills (12, 13). Lygnugaryte-Griksiene et al. examined the factors that affect the suicide intervention skills of emergency workers in Lithuania, reporting that workers’ attitudes changed as knowledge was acquired (14, 15). When education and training were provided to emergency medical services personnel, including paramedics, changes in attitudes towards suicide were observed in paramedics aged 55 and over, and paramedics with higher levels of suicide intervention skills showed more positive attitudes towards suicide prevention (15). Additionally, several previous studies reported that various types of experience can change attitudes towards suicide. Bogata et al. reported that nursing professionals with experience caring for patients at risk of suicide were less likely to blame the patient for their suicide compared with those without such experience (16). Another study examined the attitudes of social workers involved in psychosocial support for people who have attempted suicide, and the factors that influence these attitudes (17). The results revealed that social workers who had experience working with people who had attempted suicide, were suicidal, or felt remorse, tended not to think that suicide was not unjustified (17). Additionally, social workers who had personal acquaintances who had previously attempted suicide, or who had been in contact with people who had suicidal thoughts, tended to see suicide as a more common occurrence, did not think that people who had expressed their intention to commit suicide were unlikely to go through with it, and did not see suicide as an act that could not be justified (17).

Suicide prevention should be carried out by a range of frontline personnel in interpersonal support roles, who come into contact with people who have attempted suicide and provide support (18). However, as seen in a previous study, numerous factors influence the knowledge, attitudes, and behaviors of human service professionals. Yet, few studies have compared the factors affecting knowledge, attitudes, and behaviors regarding suicide across different interpersonal support workers (19). The authors hypothesized that these factors differ across support professions, creating gaps between them. They further posited that identifying and bridging these gaps could contribute to establishing a foundation for modelling future suicide prevention education and strategies.

This study primarily aimed to clarify the attitudes and influencing factors of paramedics, nurses, and social workers. The roles of paramedics, nurses, and social workers towards suicide attempters differ depending on the time and situation. Paramedics encounter suicide attempters just after they have injured themselves, often in chaotic scenes. Nurses typically care for the patient at the start of treatment after a suicide attempt. In contrast, social workers support the patient after acute-phase treatment, through consultation about lifestyle issues. The current study is expected to provide findings for collaboration among these different professions to work together to deliver effective care for suicide attempters.

During this study, the COVID-19 pandemic occurred. Given that changes in the prevalence of depression and suicide rates were observed globally both before and after this infectious disease outbreak (20–22), this study also examined the impact of the pandemic.

## 2 Methods

### 2.1 Study design

The study design was a cross-sectional study. We investigated the attitudes of fire and emergency services personnel (Paramedic Group), nurses (Nurse Group), and social workers (SW Group) towards suicide. Additionally, we investigated the backgrounds of participants and the impacts of coronavirus disease 2019 (COVID-19) on the outcomes.

### 2.2 Setting

We conducted this survey at a suicide prevention training session. The participants were paramedics, nurses and social workers who had attended training sessions on suicide prevention held by the authors in various parts of Japan. In Japan, a surge in the number of suicides occurred in 1998 (32,863 victims compared with 24,391 in 1997), and stayed at an extremely high level for 14 years (23). In response, The General Principles of Suicide Prevention in Japan was published in 2007 by The Basic Act on Suicide Prevention (24). Recruiting and training personnel engaged in suicide countermeasures is one of the priorities described in The General Principles of Suicide Prevention (24). Accordingly, educational workshops on suicide prevention for healthcare professionals are commonly held in communities. Each participant of the present study took part in one of these workshops in their respective community.

The participants filled in the questionnaires before the training sessions on suicide prevention began.

### 2.3 Data source

The research subjects (in total, 861) were all participants in the training sessions indicated above. The survey period was between 2015 and 2022. The survey was conducted using paper-based questionnaires and self-administered questionnaires.

The survey forms and self-administered questionnaires were distributed before the start of the training session, and participants were asked to complete them before the training session began. Then the questionnaires were collected by the researchers or designated staff.

### 2.4 Variable

The survey form for the questionnaire asked participants to provide information about their current occupation (social worker, paramedic, nurse), before (until 2019) and after (beyond 2020) the COVID-19 pandemic, gender (male or female), age (four age groups: 20–29 years, 30–39 years, 40–49 years, and over 50 years), presence or absence of experience caring for suicidal patients in the past year (with or without experience), presence or absence of suicide prevention training (with or without training), and the number of years they had been working in their current occupation (per 1 year increase). These variables were determined on the basis of previous studies (14, 17). Although COVID-19 was not adopted as a variable in previous studies, it was adopted as a variable in this study because it affects suicide prevention (25).

### 2.5 Outcome

The self-administered questionnaire was based on the Japanese version of the Attitudes Toward Suicide (ATTS) scale (13). ATTS is a self-administered rating scale (4). The outcome measure of this study is the ATTS score.

The ATTS scale was developed in Sweden by Renberg et al. to assess individuals’ attitudes towards suicide (4). The ATTS can be utilized in large survey studies and is considered to be the most feasible scale for assessing attitudes toward suicide (26). The ATTS makes it possible to examine similarities and differences of attitudes among other professions in the future. The factor structure of the ATTS among Japanese individuals was clarified by Kodaka et al. in a study targeting Japanese social workers (13). This study demonstrated six factors (37 items), and based on this research, the original Japanese version of the questionnaire (13) was developed. This study adopted the original Japanese version of the questionnaire used in the previous study. The ATTS comprises the following 6 factors and 37 items, consistent with the previous study: “Preventability/Readiness to help” (items 1, 30, 37), “Unjustified Behavior” (items 2, 3), “Common Occurrence” (items 14, 15, 17, 28, 31), “Suicidal expression as mere threat” (items 12, 33), “Right to Suicide” (items 5, 16, 29, 32, 34, 36), and “Impulsiveness” (items 4, 10, 22), which were assessed using a 5-point Likert scale ranging from “strongly disagree: 5” to “strongly agree: 1”. Higher scores for “Preventability/Readiness to help” indicate a positive attitude towards suicide prevention and a readiness to help individuals at risk of suicide. Higher scores for “Unjustified Behavior” indicate a stronger belief that suicide is a bad and unjustifiable act. Higher scores for “Common Occurrence” indicate a stronger view that suicide is common and normal. Higher scores for “Suicidal expression as mere threat” indicate a stronger belief that people who talk about suicide will not actually take their own lives. Lower scores for “Right to Suicide” indicate stronger agreement with the right to suicide. Lower scores for “Impulsiveness” indicate a stronger tendency to regard suicide as an impulsive act. Permission to use the scale was obtained from the original authors.

### 2.6 Statistical method

The main comparison in this study was between respondents with different occupations. As a secondary objective, we examined changes before and after the onset of the COVID-19 pandemic. All of the variables mentioned above were set as confounding factors. The evaluation method used simple and multiple linear regression models. In the simple and multiple linear regression analysis, the six ATTS factors (“Preventability/Readiness to help”, “Unjustified Behavior”, “Common Occurrence”, “Suicidal expression as mere threat”, “Right to Suicide” and “Impulsiveness”) as the response variables, and respondent’s current occupation (social worker, paramedic, nurse), COVID-19 (before [up to 2019] or after [from 2020 onwards]), gender (male or female), age (three groups: 20–29 years, 30–39 years, 40–49 years), presence or absence of experience caring for suicidal patients in the past year (without experience or with experience), presence or absence of suicide prevention training (without training or with training), and the number of years respondents had been working in their current occupation (per 1 year increase) were used as explanatory variables. In the multiple linear regression analysis, the explanatory variables were assigned using the forced-entry method. Additionally, several indicators of model fit, such as R² were reported. We calculated Cronbach’s alpha for each subscale of the ATTS. The estimated value is represented as “β” and the 95% confidence interval is represented as “95% CI”. This study is an exploratory observational study. P-values are reported for supplementary nature. All statistical analyses were performed using JMP Pro 17.0. All data from individuals with missing values were excluded, and statistical evaluation was performed on the data of the remaining participants.

This study was conducted with the approval of the Sapporo Medical University Ethics Committee (Approval No. 3-1-23).

## 3 Results

### 3.1 Participants’ characteristics

**Figure 1:**
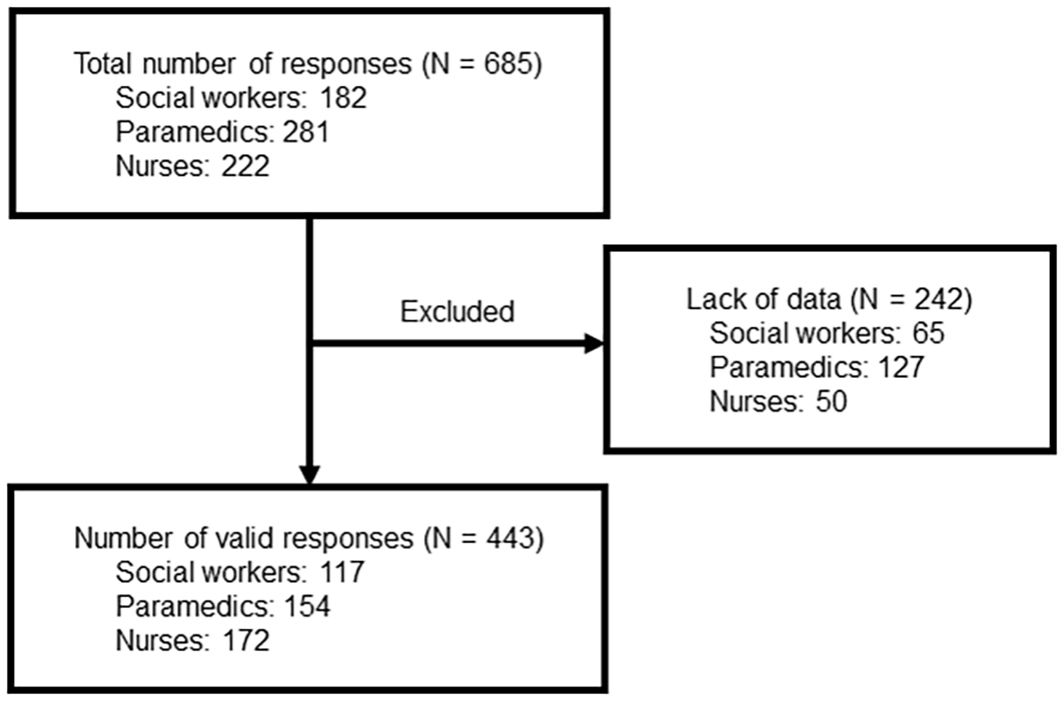
Flow of participants.

The total number of responses to the questionnaire was 685. There were 443 valid responses (154 [34.8%] in the Paramedic Group, 172 [38.8%] in the Nurse Group, and 117 [26.4%] in the SW Group, **Figure 1**).

The participants were classified according to their current occupation (**Table 1**). The gender breakdown was as follows: 222 (50.1%) male participants (152 in the Paramedic Group, 18 in the Nurse Group, and 52 in the SW Group). There were 221 (49.9%) female participants (two in the Paramedic Group, 154 in the Nurse Group, and 65 in the SW Group).

**Table 1:** Participants’ characteristics.

	Variables	Current occupation		
		Social Worker (N = 117)	Paramedic (N = 154)	Nurse (N = 172)
COVID-19	Before (until 2019)	115 (98.3)	106 (68.8)	171 (99.4)
	After (after 2020)	2 (1.7)	48(31.2)	1 (0.6)
Gender	Male	52 (44.4%)	152 (98.7%)	18 (10.5%)
	Female	65 (55.6%)	2 (1.3%)	154 (89.5%)
Age	20-29	30 (25.6%)	121 (78.6%)	19 (11.0%)
	30-39	45 (38.5%)	22 (14.3%)	25 (14.5%)
	40-49	22 (18.8%)	8 (5.2%)	70 (40.7%)
	≥ 50	20 (17.1%)	3 (1.9%)	58 (33.7%)
Experience caring for suicidal patients in the past year	Without experience	21 (17.9%)	100 (64.9%)	82 (47.7%)
	With experience	96 (82.1%)	54 (35.1%)	90 (52.3%)
History of participation in suicide prevention training	Without training	50 (42.7%)	143 (92.9%)	137 (79.7%)
	With training	67 (57.3%)	11 (7.1%)	35 (20.3%)
Number of years working in current occupation	0-2	14 (12.0%)	44 (28.6%)	18 (10.5%)
	3-5	27 (23.1%)	62 (40.3%)	13 (7.6%)
	6-10	34 (29.1%)	29 (18.8%)	19 (11.0%)
	11-15	18 (15.4%)	13 (8.4%)	17 (9.9%)
	16-20	12 (10.3%)	3 (1.9%)	28 (16.3%)
	≥ 21	12 (10.3%)	3 (1.9%)	77 (44.8%)
	Average ± SD (years)	10.1 ± 7.7	5.5 ± 5.4	18.4 ± 10.7

Participants’ age distribution was as follows: 170 (38.4%) participants were in their 20s (121 in the Paramedic Group, 19 in the Nurse Group, and 30 in the SW Group); 92 (20.8%) participants were in their 30s (22 in the Paramedic Group, 25 in the Nurse Group, and 45 in the SW Group); 100 (22.6%) participants were in their 40s (eight in the Paramedic Group, 70 in the Nurse Group, and 22 in the SW Group); 81 (18.3%) participants were aged 50 and over (three in the Paramedic Group, 58 in the Nurse Group, and 20 in the SW Group).

The details of participants’ experience dealing with suicides before and after the spread of COVID-19, presence or absence of a history of suicide prevention training, and number of years working in current occupation are shown in **Table 1**.

### 3.2 Overall ATTS results

Cronbach’s alpha of subscale scores was 0.46 for “Preventability/Readiness to help”, 0.66 for “Unjustified Behavior”, 0.55 for “Common Occurrence”, 0.75 for “Suicidal expression as mere threat”, 0.71 for “Right to Suicide”, and 0.26 for “Impulsiveness”.

The results revealed that the average score for “Preventability/Readiness to help” was 3.38 (SD: 0.660), and participants exhibited a tendency to think that “suicide prevention is possible” (**Table 2**).

**Table 2:** Descriptive statistics for all participants in ATTS.

	Average	SD	Median
Preventability/readiness to help	3.38	0.660	3.33
Unjustified behavior	3.56	0.907	3.50
Common occurrence	3.01	0.679	3.00
Suicidal expression as mere threat	2.68	0.922	3.50
Right to suicide	2.55	0.685	3.00
Impulsiveness	2.87	0.665	3.00

The average score for “Unjustified Behavior” was 3.56 (SD: 0.907), indicating a positive attitude towards the statement “Suicide is an unjustified act” (**Table 2**).

The average score for “Common Occurrence” was 3.01 (SD: 0.679), indicating a tendency to think that “Suicide can happen to anyone” (**Table 2**).

The average score for “Suicidal expression as mere threat” was 2.68 (SD: 0.922), indicating a negative attitude towards the statement “People who hint at suicide do not commit suicide” (**Table 2**).

The average score for “Right to Suicide” was 2.55 (SD: 0.685), indicating a negative attitude towards the notion of a “Right to Suicide” (**Table 2**).

The average score for “impulsiveness” was 2.87 (SD: 0.665), indicating a negative attitude towards the idea that “suicide is impulsive” (**Table 2**).

The details by occupation, before and after the spread of COVID-19, gender, age group, experience of dealing with suicides, and presence or absence of history of suicide prevention training are shown in **Supplementary 1**.

### 3.3 Analysis of linear regression model results

The results of the multiple linear regression analysis are shown in **Table 3** . The results are shown for each subcategory of ATTS.

For “Preventability/Readiness to help”, the results were as follows: participants in their 30s (β = −0.220, 95% CI: −0.414–−0.026, P = 0.026), 40s (β = −0.306, 95% CI: −0.550–−0.063, P = 0.014), and those 50 and over (β = −0.293, 95% CI: −0.584–−0.001, P = 0.049) scored significantly lower than participants in their 20s. In addition, scores were significantly higher for participants who had experience caring for suicidal patients in the past year (β = 0.193, 95% CI: 0.060–0.326, P = 0.005) compared with those who did not have experience caring for suicidal patients in the past year (**Table 3-1**).

In the “Unjustified Behavior” category, the Paramedic Group (β = 0.474, 95% CI: 0.167–0.780, P = 0.003) scored significantly higher than the SW Group. In addition, the scores of participants in their 40s (β = 0.388, 95% CI: 0.060–0.716, P = 0.020) and those who were 50 and over (β = 0.485, 95% CI: 0.092–0.878, P = 0.016) were significantly higher than those of participants in their 20s (**Table 3-1**).

In the “Common Occurrence” category, the Paramedic Group (β = −0.640, 95% CI: −0.855–−0.425, P < 0.001) scored significantly lower than the SW Group. In addition, scores for participants with “experience caring for suicidal patients in the past year” (β = 0.140, 95% CI: 0.015–0.265, P = 0.029) were significantly higher than those for participants with “no experience caring for suicidal patients in the past year” (**Table 3-1**).

**Table 3-1:** Results of multiple regression analysis.

		Preventability/readiness to help				Unjustified behavior				Common occurrence			
	Model of fit	R^2^ = 0.057				R^2^ = 0.095				R^2^ = 0.208			
		β	95% CI		P-value	β	95% CI		P-value	β	95% CI		P-value
Current occupation													
	Social worker	Reference				Reference				Reference			
	Paramedic	-0.212	-0.439	0.016	0.069	0.474	0.167	0.780	0.003	-0.640	-0.855	-0.425	< 0.001
	Nurse	-0.091	-0.284	0.101	0.350	0.135	-0.123	0.394	0.304	-0.105	-0.286	0.076	0.255
COVID-19													
	Before (until 2019)	Reference				Reference				Reference			
	After (beyond 2020)	0.070	-0.147	0.286	0.527	0.175	-0.116	0.466	0.238	-0.079	-0.283	0.125	0.448
Gender													
	Male	Reference				Reference				Reference			
	Female	-0.039	-0.227	0.150	0.689	-0.108	-0.362	0.147	0.405	-0.010	-0.188	0.168	0.913
Age
	20-29	Reference				Reference				Reference			
	30-39	-0.220	-0.414	-0.026	0.026	0.066	-0.195	0.327	0.620	-0.013	-0.196	0.171	0.893
	40-49	-0.306	-0.550	-0.063	0.014	0.388	0.060	0.716	0.020	-0.115	-0.345	0.115	0.327
	≥ 50	-0.293	-0.584	-0.001	0.049	0.485	0.092	0.878	0.016	0.019	-0.256	0.294	0.893
Experience caring for suicidal patients in the past year													
	Without experience	Reference				Reference				Reference			
	With experience	0.193	0.060	0.326	0.005	-0.137	-0.315	0.042	0.134	0.140	0.015	0.265	0.029
History of participation in suicide prevention training													
	Without training	Reference				Reference				Reference			
	With training	0.031	-0.128	0.191	0.699	-0.107	-0.322	0.108	0.327	0.090	-0.060	0.241	0.240
Number of years working in current occupation													
	per 1 year increase	0.003	-0.008	0.013	0.626	-0.008	-0.022	0.006	0.268	-0.006	-0.015	0.004	0.261

In the “Suicidal expression as mere threat” category, the Paramedic Group (β = 0.450, 95% CI: 0.142–0.757, P = 0.004) and the Nurse Group (β = 0.394, 95% CI: 0.134– 0.653, P = 0.003) had significantly higher scores than the SW Group. Additionally, participants with suicide prevention training experience (β = −0.234, 95% CI: −0.450–−0.019, P = 0.033) scored lower than participants without suicide prevention training experience (**Table 3-2**).

**Table 3-2:** Results of multiple regression analysis.

		Suicidal expression as mere threat				Right to suicide				Impulsiveness			
	Model of fit	R^2^ = 0.120				R^2^ = 0.032				R^2^ = 0.034			
	Variables	β	95% CI		P-value	β	95% CI		P-value	β	95% CI		P-value
Current occupation													
	Social worker	Reference				Reference				Reference			
	Paramedic	0.450	0.142	0.757	0.004	0.055	-0.185	0.295	0.652	0.169	-0.063	0.401	0.154
	Nurse	0.394	0.134	0.653	0.003	0.095	-0.107	0.297	0.356	0.220	0.024	0.416	0.028
COVID-19													
	Before (until 2019)	Reference				Reference				Reference			
	After (beyond 2020)	-0.153	-0.445	0.139	0.305	-0.050	-0.277	0.177	0.665	0.068	-0.152	0.289	0.542
Gender													
	Male	Reference				Reference				Reference			
	Female	-0.114	-0.369	0.141	0.380	0.016	-0.183	0.214	0.877	0.003	-0.190	0.195	0.980
Age													
	20-29	Reference				Reference				Reference			
	30-39	-0.131	-0.393	0.131	0.326	-0.009	-0.213	0.195	0.934	0.148	-0.050	0.346	0.142
	40-49	0.027	-0.302	0.356	0.870	-0.220	-0.476	0.036	0.092	-0.057	-0.305	0.192	0.655
	≥ 50	0.107	-0.287	0.501	0.594	-0.136	-0.443	0.170	0.383	0.094	-0.203	0.392	0.534
Experience caring for suicidal patients in the past year													
	Without experience	Reference				Reference				Reference			
	With experience	-0.160	-0.339	0.019	0.080	-0.101	-0.241	0.038	0.155	0.110	-0.026	0.245	0.113
History of participation in suicide prevention training													
	Without training	Reference				Reference				Reference			
	With training	-0.234	-0.450	-0.019	0.033	-0.083	-0.251	0.084	0.329	-0.019	-0.182	0.144	0.818
Number of years working in current occupation													
	per 1 year increase	-0.008	-0.022	0.005	0.233	0.000	-0.011	0.011	0.973	0.004	-0.007	0.014	0.469

There were no significant differences in the “Right to Suicide” items.

In the “Impulsiveness” items, the Nurse Group (β = 0.220, 95% CI: 0.024–0.416, P = 0.028) scored significantly higher than the SW Group (**Table 3-2**).

Comparison between the nurse group and the paramedic group. “Preventability/Readiness to help” was β = −0.120, 95% CI: −0.373–0.132, P = 0.350; “Unjustified Behavior” was β = 0.338, 95% CI: −0.002–0.678, P = 0.051, “Common Occurrence” was β = −0.534, 95% CI: −0.773–−0.296, P < 0.001, “Suicidal expression as mere threat” was β = 0.056, 95% CI: −0.285–0.397, P = 0.746, “Right to Suicide” was β = −0.040, 95% CI: −0.306–0.226, P = 0.767, and “Impulsiveness” was β = −0.051, 95% CI: −0.308–0.207, P = 0.700.

The results of the simple linear regression analysis are shown in **Supplementary 2**.

## 4 Discussion

The current study examined the characteristics of the attitudes of fire and emergency services personnel, nurses, and social workers towards suicide, and the factors that influence these attitudes. The results regarding the characteristics of the three professions indicated that score trends for the six items in the ATTS were similar for each profession, except for the “Common Occurrence” item, which revealed that the SW Group considered suicide to be more common than the Paramedic Group. A previous study by Kodaka et al. (17) also reported on score trends for the “Common Occurrence” item among social workers. This result indicated that the more social workers had come into close contact with people who had attempted suicide, the stronger the tendency for them to view suicide as common (17). Of the social workers who participated in the current study, 82.1% had experience dealing with suicide (**Table 1**). The Paramedic Group had a different attitude toward the SW Group regarding the “Common Occurrence” item, possibly because they considered encounters with suicide attempters to be unusual owing to the horrific circumstances, serious injuries, and chaotic scenes involved. This finding was characteristic of fire and emergency services personnel compared with participants in other occupations.

Regarding the “Unjustified Behavior” item, the Paramedic Group had a more negative attitude than the SW Group, indicating a stronger belief that suicide is unjustified. Paramedics may consider suicidal behavior to be unjustified because mental health issues of rescuers are not addressed in the standards of education and training at fire academies (27), and it is difficult for them to understand the background of suicide attempts. In addition, social workers may be more likely to be exposed to justifications for suicide through listening to the background of suicide attempts and gathering information.

The Nurse Group and Paramedic Group were less likely to agree with the notion of “Suicidal expression as mere threat” than the SW Group. This may be explained by these participants’ past participation in suicide prevention training sessions. Among the participants, 57.3% of social workers, 7.1% of paramedics, and 20.3% of nurses had previously attended suicide prevention training sessions. A previous study reported differences in attitude depending on whether or not the participants had attended a suicide prevention training session (17). Similarly, in the current study, participants who had previously attended a suicide prevention training session had more favourable attitudes towards suicide prevention.

In terms of “impulsiveness”, the SW Group had more favourable attitudes than the Nurse Group. In a previous study, participants who had attended suicide prevention training sessions were found to have more favourable attitudes (17). This change in attitudes might occur because participants learn that every suicide attempter has their own psychosocial background, mental health challenges, and risk factors for suicide at suicide prevention training sessions. Regarding actual contact with patients who have attempted suicide, the difference in attitudes between nurses and social workers may be related to the timing of contact with the patient. Nurses typically come into contact with the patient at the start of treatment after a suicide attempt, whereas social workers come into contact with the patient after acute-phase treatment, in the form of consultation about lifestyle issues. It is possible that the timing of contact has an effect on impressions of “impulsiveness”.

Differences between participants with and without experience dealing with suicides were seen in the “Common Occurrence” and “Preventability/Readiness to help” items. In a previous study, it was reported that experience dealing with suicides increases understanding of “Common Occurrence” (17), and a similar result was obtained in the current study. In that study, “Preventability/Readiness to help” was reported to be a desirable attitude for nurses with experience dealing with suicides, compared with nurses without such experience (17). In two other studies of nurses, it was reported that the nurses’ age and the number of times they had dealt with suicide also had an impact (28, 29). These results suggest that attitudes towards suicide prevention change as a result of having experience dealing with suicides.

The results revealed age-related differences in responses to the “Unjustified Behavior” and “Preventability/Readiness to help” items. Regarding the “Unjustified Behavior” item, participants in their 20s were more likely to feel that “suicide is unjustifiable” compared with those in their 40s and those aged 50 and over. Regarding the “Preventability/Readiness to help” item, participants in their 20s had a more negative attitude than those in their 30s, 40s, and 50s and above, and were more likely to feel that suicide is not preventable. Previous studies examining the effects of age on attitudes towards suicide have produced inconsistent results (24, 25, 28–36). The differences in the findings obtained in previous studies may be related to the professional backgrounds of the participants examined, as well as differences in their sociocultural background and age, and the countries in which the studies were conducted. In Sweden, it has been reported that attitudes towards suicide have changed over time (37).

The COVID-19 pandemic was reported to increase the number of people with anxiety and depressive symptoms in OECD countries such as the United States and the United Kingdom, and the number of suicides in Japan increased from 2019 to 2020 (37–39). The results of this study show that attitudes towards suicide did not change before and after the spread of COVID-19.

The current findings indicated that the Paramedic Group, Nurse Group, and SW Group had attitudes towards suicide that were common across all three professions. The three professions expressed desirable attitudes for interpersonal support workers, such as “considering suicide to be preventable”, “considering suicide to be an unreasonable action”, “not considering that people who hint at suicide will not commit suicide”, “not considering suicide to be a right”, and “considering that suicide does not occur suddenly” ( **Table 3** ).

Comparing the current results with the findings of a study by Kodaka et al. targeting social workers (17) revealed an opposite trend in scores for the “Right to Suicide” item. In the previous study (17), scores were positive regarding the “Right to Suicide” item, whereas, in the current results, all three occupations had negative scores regarding the “Right to Suicide” item. Additionally, in Kodaka et al.’s study, participants with a history of suicidal ideation were more likely to support the notion of a “Right to Suicide” than those without a history of suicidal ideation (17). However, several previous studies have suggested that considering suicide to be one’s own right may lead to a decline in suicide counselling skills (40–42). Thus, the current results may be considered to reflect a desirable attitude in suicide counselling skills or intervention skills.

In relation to suicide, Kawashima et al. reported that mental health professionals who participated in a 2-day training programme developed to provide basic learning to understand suicidal behavior and acquire skills in evidence-based suicide prevention intervention strategies working in the field of mental health reported that their attitudes towards suicide prevention, self-efficacy, skills and techniques had improved, and that they had acquired the desired attitudes towards people who had attempted suicide (43). A study in Lithuania (14) reported similar results, suggesting that participation in training workshops can change participants’ attitudes.

The current study involved several limitations that could potentially bias the results. First, the participants were limited to Japanese professionals in three occupations. The impact of this sampling limitation on the results is unclear, and caution should be exercised in generalising our findings to other countries. Given the Japanese participants, we also need to consider that we lack detailed information about their educational background during their student years. The educational content that formed the foundation of their professional practice may have influenced their work and attitudes toward patients at high risk of suicide, potentially affecting outcomes across professions. Secondly, there was a high amount of missing data. This bias may limit the generalizability of the analysis results, requiring caution in interpretation. Third, we did not investigate the work history of nurses or social workers. There may be differences in attitudes depending on whether they have experience in psychiatric or emergency medicine. Additionally, because the participants were individuals who had attended a training course, it is possible that individuals with a high level of interest in suicide were more likely to participate. Because this was a cross-sectional study, it was not possible to clarify the causal relationships between variables. Fourth, we did not investigate changes in attitudes after attending the training sessions. In the current study, we focused on investigating baseline characteristics and predictive factors. Future studies should focus on examining the effectiveness of suicide prevention training sessions. Fifth, the Cronbach’s alpha coefficients for several subscales fell below Nunnally’s conventional threshold. However, previous studies on this topic also reported low alpha coefficients (17). The reliability associated with these results is limited. Variables exploring associations with attitudes toward suicide have been examined only to a limited extent. Future research should identify other factors related to these attitudes. Finally, some of the indicators of model fit for the multiple linear regression models may not have been sufficient. If data were available, the model fit might be improved by adding more explanatory variables.

Comparing the attitudes of fire and ambulance service personnel, nurses and social workers towards suicide, participants in all three professions were found to have desirable attitudes as interpersonal support workers who come into contact with people who have attempted suicide. These findings suggest that it may be possible to implement a common suicide prevention education programme, despite some differences between the professions. The differences we observed between the professions may reflect the backgrounds, educational courses and ways of interacting with people within each profession. It is desirable to provide paramedics with education that promotes an understanding of the mental health background of suicide attempters. Identifying and bridging such interprofessional gaps among all interpersonal support workers involved—from transporting suicide attempt survivors to providing social care—could enable more effective suicide prevention education and strategies.

It may be possible to utilize the findings obtained in this study for suicide prevention education. For example, holding regional workshops bringing together different professions, conducting case studies, and deepening mutual understanding of each profession’s characteristics—such as when each profession interacts with individuals at risk of suicide and from what perspective each profession assesses such individuals—could enable seamless suicide prevention measures through collaborative care for at-risk individuals. If such an education and training program were to be developed, it would also be necessary to verify the effectiveness of this multi-professional collaboration on suicide prevention.

## Data Availability

The datasets presented in this article are not readily available because we have no consent from participants to use this as a dataset. Requests to access the datasets should be directed to Tomonori Kashiwagi, t.kashiwa19860602@gmail.com.
